# A pilot study of the impact of enhanced cardiac index coupled with pulsatile flow on goal direct perfusion during cardiopulmonary bypass

**DOI:** 10.1051/ject/2025060

**Published:** 2026-06-19

**Authors:** Mostafa Bagherinasab, Sahra Rezaei, Ali Reza Moradi, Bagher Aghal, Julie Steele-Pruett, Nathaniel H. Darban

**Affiliations:** 1 Extracorporeal Life Support, Baqiyatallah University of Medical Sciences Tehran Iran; 2 Tehran University of Medical Sciences Tehran Iran; 3 Baqiyatallah University of Medical Sciences Tehran Iran; 4 Midwestern University, College of Health Sciences, Cardiovascular Science Program 19555 N. 59TH Avenue Glendale AZ 85308 USA

**Keywords:** Cardiac index, Pulsatile flow, Cardiopulmonary bypass, Organ perfusion

## Abstract

*Introduction*: Providing sufficient organ perfusion during cardiopulmonary bypass (CPB) is a common research topic among extracorporeal technology researchers. The application of an elevated cardiac index (CI) to enhance CPB flow, coupled with the adoption of pulsatile flow, constitutes a novel approach designed to improve organ perfusion and oxygen delivery (DO2). this study aims to assess the effects of increased CI and pulsatile flow on organ perfusion during CPB. *Material and methods*: In this pilot study, thirty patients scheduled for on-pump coronary artery bypass graft (CABG) surgery with an estimated prolonged CPB time were enrolled. Patients were randomly divided into two study groups. Patients in the control group were managed with a CI of 2.4 L/min/m^2^, while patients in the study group received a CI equal to 2.6 to 3 L/min/m^2^ with pulsatile flow (PF) throughout the bypass run. Lactate fluctuations, creatinine variation, inotrope needs, blood transfusion requirements, ICU and hospital length of stay were assessed and noted. *Results*: Participants in the study group exhibited lower creatinine levels throughout the assessment period; however, this difference did not reach statistical significance (*P* > 0.05). Participants in the study group consistently exhibited significantly lower lactate concentrations over the course of the investigation (*P* < 0.05). Patients in the study groups experienced a reduced duration of both ICU and hospital lengths of stay; however, this difference did not reach statistical significance (*P* > 0.05). *Conclusion*: This prospective study concludes that an increased CI in conjunction with PF during CPB can markedly enhance organ perfusion, as evidenced by a statistically significant reduction in lactate production observed throughout the duration of the bypass.

## Introduction

Over a million cardiac surgeries are conducted globally on an annual basis [1]. The majority of these procedures are performed using cardiopulmonary bypass (CPB), allowing surgeons to operate on a bloodless field on a quiescent heart, while ensuring continuous perfusion and oxygenation. The utilization of CPB carries inherent risks and is frequently linked to postoperative organ dysfunction [1, 2]. A variety of retrospective studies indicate that the relationship between significant hemodilution during CPB and inadequate organ perfusion may be attributed to a deficiency in oxygen delivery (DO2) [3, 4].

The notion that pump flow should be modulated in accordance with the DO2 instead of relying solely on body surface area and a cardiac index (CI) ranging from 1.8 to 2.4 L/min/m^2^ is a prevalent topic of discussion among scholars [3]. Currently, there exists a lack of robust evidence indicating that a goal-directed perfusion (GDP) approach aimed at preventing DO2 from falling below a critical threshold will effectively diminish the incidence of ischemia in vital organs during CPB [5].

Significant endeavours have been dedicated to the identification of strategies that protect organs, and within these efforts, pulsatile flow (PF) and adjustments in blood flow during CPB have emerged as valuable parameters [1, 3].

Roller and centrifugal pumps are commonly utilized to generate non-pulsatile flow (NPF), which remains the predominant mode of perfusion [6]. However, it is also feasible to imitate the arterial pulse produced by the heart, which is believed to be more in line with physiological processes [7]. Pulsatile flow can be created in the CPB system by utilizing pulsator devices in the circuit or by adjusting the arterial pump to produce rapid changes in flow [6, 8]. There is substantial evidence supporting the role of PF in reducing the systemic inflammatory response and safeguarding the function of vital organs such as the kidneys, brain, lungs, blood, and heart [8]. The 2019 guidelines on CPB in adult cardiac surgery, jointly issued by the European Association for Cardio-Thoracic Surgery (EACTS), European Association of Cardiothoracic Anaesthesiology (EACTA), and European Certificate in Cardiovascular Perfusion (EBCP), suggest that patients at high risk of adverse lung and renal outcomes should be considered for pulsatile perfusion [9]. The Energy Equivalent Pressure (EEP) is a recognized metric utilized to evaluate the effectiveness of pulsatile flow. It has been established that an EEP value exceeding 15% of the mean arterial pressure indicates an adequate pulsatile flow that can effectively replicate physiological pulsatility [8].

Recent research has provided evidence that the notion of GDP, aimed at sustaining the minimum DO2 level above the critical threshold, is an additional contributing factor to inadequate perfusion during CPB [1]. The modifiable factors in the delivery of DO2 formula can be adjusted by changes in CI, thereby affecting the CPB flow. Research indicates that an elevation in CI to levels as high as 3 L/min/m^2^ can markedly enhance organ perfusion, which in turn may lead to improved outcomes for patients undergoing cardiopulmonary bypass surgery [5].

In this research, our objective is to evaluate the impact of incorporating PF and an increased cardiac index on organ perfusion during CPB.

## Materials and methods

### Patient characteristics

In this pilot study, we carried out a comparative study between the conventional management of CPB flow and a combination of PF and higher CI to assess organ perfusion.

The research protocol was reviewed and approved by the Institutional Review Board at Baqiyatallah University of Medical Sciences. This approval includes the right to publish the findings. The research protocol was evaluated and approved by the Institutional Review Board at Baqyiatallah University of Medical Sciences. It was classified as a low-risk study that met the criteria for waiving individual consent, as specified in the institutional guidelines. Thirty patients who underwent coronary artery bypass graft (CABG) surgery with long CPB time at our medical facility were assessed and divided into two distinct study groups.

The eligibility requirements consisted of (1) being 18 years of age or older, (2) having a left ventricular ejection fraction of 50% or higher, (3) having normal preoperative serum creatinine levels (60 to 105 μmol/L for men, 45 to 90 μmol/L for women), (4) undergoing elective cardiac surgery with prolonged CPB time, and (5) maintaining a body temperature between 33 and 35 °C during CPB. Exclusion criteria included: (1) non-elective surgery, (2) body mass index greater than or equal to 32 kg/m^2^, (3) history of cerebrovascular disease, (4) Abnormal plasma lactate levels (>2 mmol/L) prior to CPB initiation, (5) uncompensated diabetes, (6) autoimmune disease, (7) active infection, (8) any immunosuppressant therapy, and (9) coagulation disorder.

### Anesthesia management

Anesthesia was induced using fentanyl (5–10 μg/kg), Cisatracurium (0.15–0.2 mg/kg), propofol (1–1.5 mg/kg), and Midazolam (70–80 μg/kg) and then maintained with intravenous anesthesia (fentanyl 1.5 mg, Atracurium 150 mg, Midazolam 15 mg) throughout the surgical procedure. Norepinephrine (20 μg/cc) was utilized as a bolus dose to sustain a mean arterial pressure (MAP) greater than 60 mmHg throughout the case. The utilization of inotropes during the surgery was noted.

### Conduct of CPB

The CPB system included an Inspire 8F oxygenator (LivaNova, Mirandola, Italy) and an Inspire hard-shell venous reservoir (LivaNova). The equipment comprised a Stöckert S5 heart–lung machine with roller pump (LivaNova) and a Stöckert Heater Cooler System 3T (LivaNova). The priming solution was composed of 1,500 mL of Ringer’s solution and 10,000 IU heparin. Additionally, 200 mL of 20% mannitol and 100 mL of 20% albumin were included in the CPB prime.

Following heparinization at a dose of 400 IU/kg, the aortic root was cannulated, followed by bicaval venous cannulation based on the specific surgical procedure. To accommodate high CI and PF without exceeding safe arterial line pressures, we used arterial cannula ranging from 20 Fr to 24 Fr depending on patient BSA, sourced from LivaNova (Italy). Cannula selection followed the manufacturer's flow-pressure charts to maintain a pressure gradient below 80 mmHg. We did not encounter excessive arterial pressures (>300 mmHg) even in patients with BSA > 2.0. Our surgical team was supportive of using a larger cannula when clinically justified.

The activated clotting time was maintained at a level exceeding 480 seconds throughout the CPB procedure. All patients received active mild hypothermia during CPB (33 °C–35 °C). All individuals were administered 1250 mL of Del Nido cardioplegia (DN), consisting of a mixture of 1 part oxygenated blood to 4 parts DN crystalloid solution.

Every 90 min, a maintenance dose of 625 mL of 1:4 DN cardioplegia was administered. An alpha-stat blood gas management strategy was implemented throughout CPB. Following weaning from CPB, protamine sulfate (4 mg/kg) was administered to reverse the effects of heparin.

### Study protocol

CPB was initiated with a baseline flow rate of 2.4 L/min/m^2^ for every participant. The study began after achieving stable hemodynamic conditions after placement of the aortic cross-clamp and cardioplegia administration. A total of thirty patients were randomly allocated to two distinct study groups.

Patients in the control group (*n* = 15) were provided with a non-pulsatile flow rate of 2.4 L/min/m^2^ continuously during the surgical procedure. If hypotension occurred during the CPB period, Norepinephrine was given as a bolus to elevate MAP above 60 mmHg.

Patients in the study group (*n* = 15) were administered a pulsatile flow rate ranging from 2.6 to 3 L/min/m^2^ during the entire duration of the surgery. The pulsatile configuration was defined by a baseline flow rate of 30%, a width of 60%, and a frequency of 70 beats per minute. The quality of the pulsatile flow was evaluated through the use of Energy Equivalent Pressure (EEP). In instances where the EEP was determined to be equal to or less than the Mean Arterial Pressure (MAP), adjustments were made by increasing the width and frequency parameters in order to achieve an EEP that surpassed the MAP by 15% [8].

If hypotension occurred during the CPB period, the flow rate was initially increased to 3 L/min/m^2^, and if hypotension persisted, Norepinephrine was utilized.

The venous reservoir volume was maintained above 10% of the CPB flow rate (L/min), and crystalloid solution (Ringer’s acetate) was administered into the reservoir to achieve or uphold this safety threshold.

## Data collection

The preoperative information comprised patient demographic characteristics, preoperative serum creatinine levels, ventricular ejection fraction, comorbidities (such as hypertension or chronic obstructive pulmonary disease), and initial haemoglobin (Hb).

Perioperative information encompassed the duration of CPB, time of aortic clamp, haematocrit (Hct) and Hb values (measured at the initiation of CPB and every half hour thereafter), urine output, requirement for inotropic drugs, mean value of oxygen delivery index (DO2i), serum lactate levels, and blood transfusions during CPB. Postoperative data comprised serum creatinine levels, serum lactate levels, inotropic agent needs, blood transfusions, and length of stay in the intensive care unit (ICU) and hospital.

The calculation of DO2i was performed using the following formula: DO2i (mL/min/m2) = pump flow (L/min) × [Hct/2.94 (g/dL) × 1.36 × arterial oxygen saturation (%) + partial pressure of arterial oxygen (mm Hg) × 0.003] × 10/BSA (m2). The B-Capta online blood gas monitoring system developed by LivaNova was employed, facilitating precise measurements of partial pressure of oxygen (pO2) and temperature within the arterial line, as well as saturation, HCT, Hb, and temperature in the venous line. The calculation of DO2i was performed manually at 15-minute intervals, utilizing the specified formula.

The primary endpoints were serum lactate level during CPB and post-surgery, nadir DO2i, and perioperative urine output.

Secondary end points were postoperative serum creatinine levels, blood transfusion requirements, and length of ICU and hospital stay.

### Statistical analysis

All data are expressed as mean ± standard error of the mean or as absolute numbers and percentages, as appropriate. Statistical analysis was performed using SPSS version 11.0 software (SPSS Inc., Chicago, Ill). An independent *t*-test was utilized to assess the differences in means for quantitative variables, while a Chi-square test was applied to evaluate the relationships among qualitative variables. Repeated measures analysis of variance, accompanied by Mauchly’s test of sphericity, was employed to assess the variation of DO2i across different time intervals.

## Results

Demographic and preoperative details of the patient population are shown in Tables 1 and 2. The analysis revealed that all demographic and pre-operative variables were comparable, with the exception of EF, which was found to be significantly lower in the study group.

**Table 1:** Preoperative profile.

Characteristic	Study group (*n* = 15)	Control group (n = 15)	*P* value
Age(year)	61.6 ± 11.31	60.53 ± 11.08	0.79
Male *n* (%)	10 (66.7)	9 (60%)	0.70
BSA	1.89 ± 0.22	1.75 ± 0.20	0.09
EF (%)	42.33 ± 9.42	48.33 ± 5.56	0.04

Values are presented as mean ± standard deviation, or *n* (%).

**Table 2:** Participants comorbidity disease.

Parameter	Study group (*n* = 15)	Control group (*n* = 15)	*P* value
Opium addiction *n* (%)	4 (26.7%)	1 (6.7%)	0.33
CVA *n* (%)	1 (6.7%)	0	0.50
CRF *n* (%)	3 (20%)	1 (6.7%)	0.59
Carotid stenosis more than 60% *n* (%)	3 (20%)	1 (6.7%)	0.59
DM *n* (%)	1 (6.7%)	3 (20%)	0.59
HTN *n* (%)	5 (33.3%)	6 (40%)	0.16

Values are presented as *n* (%). CVA, cerebral vascular accident; CRF, Chronic renal failure; DM, Diabetes mellitus; HTN, Hypertension.

The peri-operative data presented in Table 3 indicate that patients within the study group exhibit a significantly increased urine output, a reduced ultrafiltration rate, and a lower requirement for inotropic support. The mean Do2 value in the study group was significantly higher in comparison to the control group (*P* < 0.05).

**Table 3:** Operative data.

Factor		Study group (*n* = 15)	Control group (*n* = 15)	*P* value
CPB time (min)		136.66 ± 57.67	126.46 ± 27.90	0.54
Aortic cross-clamp time (min)		112.26 ± 37.48	100.06 ± 13.45	0.25
Mean DO2i mL/min/m^2^		316.40 ± 21.25	275.06 ± 11.44	<.001
Urine output (mL)		760.0 ± 399.64	506.66 ± 215.26	0.03
Ultrafiltration (mL)		1666.66 ± 951.44	2360.0 ± 479.28	0.02
Inotrope requirement (μg)		110.0 ± 78.19	281.33 ± 171.25	0.002
RBC transfusion *n* (%)	1 unite	6 (40%)	7 (46.7%)	0.21
	2 unite	0	1 (6.7%)	0.10

Values are presented as mean ± standard deviation, or *n* (%). CPB, Cardiopulmonary Bypass; DO2i, delivery of oxygen.

Table 4 illustrates the variations in creatinine levels between the study groups. Throughout the evaluation period, individuals in the study group exhibited lower levels of creatinine, with a significant decline occurring right after the surgery and continuing at the 24 and 48-hour marks post-surgery.

**Table 4:** Pre and post-operative creatinine variation.

Variable	Study group (*n* = 15)	Control group (*n* = 15)	*P* value
Creatinine mg/dL (base)	1.34 ± 0.61	1.04 ± 0.25	0.08
Creatinine mg/dL (after surgery)	1.38 ± 0.60	1.86 ± 0.45	0.02
Creatinine mg/dL (12 h after surgery)	1.25 ± 0.58	1.44 ± 0.34	0.28
Creatinine mg/dL (24 h after surgery)	1.20 ± 0.52	1.75 ± 0.34	0.002
Creatinine mg/dL (48 h after surgery)	1 ± 0.33	1.52 ± 0.17	<.001

Values are presented as mean ± standard deviation.

Table 5 illustrates the differences in lactate levels between the study groups, revealing that participants in the study group consistently exhibited significantly lower lactate concentrations over the course of the investigation (*p* < .001).

**Table 5:** Pre and post-operative lactate variation.

Variable	Study group (*n* = 15)	Control group (*n* = 15)	*P* value
Lactate (base) (mmol/L)	0.91 ± 0.28	1.05 ± 0.41	0.29
Lactate 1 (mmol/L)	0.94 ± 0.35	2.77 ± 0.97	<.001
Lactate 2 (mmol/L)	1.12 ± 0.27	4.54 ± 1.63	<.001
Lactate 3 (mmol/L)	1.59 ± 0.39	5.45 ± 1.56	<.001
Lactate 4 (mmol/L)	1.22 ± 0.38	4.86 ± 2.51	<.001
Lactate 5 (mmol/L)	0.99 ± 0.38	3.58 ± 2.06	<.001

Values are presented as mean ± standard deviation. Lactate 1, 30 min after aortic cross clamp, lactate 2, 60 min after Aortic cross clamp, lactate 3, immediately after aortic de-clamp, lactate 4, 6 h after ICU admission and lactate 5, 12 h after ICU admission.

The post-operative data presented in Table 6 indicate that patients in the study groups experienced a significant reduction in both ICU and hospital stays (*p* < 0.05).

**Table 6:** Post-operative profile.

Parameter	Study group (*n* = 15)	Control group (*n* = 15)	*P* value
ICU stay (day)	3.93 ± 1.22	5.33 ± 1.04	0.002
Hospital stay (day)	8.06 ± 2.18	11.66 ± 1.79	<.001

## Discussion

This study primarily aimed to investigate the correlation between various perfusion flow techniques and variations in lactate levels as indicators of organ perfusion. In addition, we evaluated several additional factors, including fluctuations in creatinine levels, urine output, the necessity for intraoperative inotropes, the requirement for blood transfusions, and the duration of both ICU and hospital lengths of stay.

The constraints associated with lactate as a strong predictive indicator center on the notion that hyperlactatemia could result from a confluence of both intraoperative and postoperative influences [10]. The relationship between reduced flow rates (less than 100 mL/kg/min) and DO2 during CPB, along with the duration of CPB, circulatory arrest, HCT levels on CPB, temperature during and after CPB, and the systemic inflammatory responses, has been demonstrated to correlate with intra- and postoperative lactate levels [11, 12]. This research indicated that the patients within the study group exhibited elevated levels of DO2 during CPB. While certain studies suggest a direct correlation between increased DO2 and reduced lactate levels [5, 13], other investigations have demonstrated that no such direct relationship exists between elevated DO2 and lactate production during CPB [14, 15].

The ability of DO2i to predict adequate organ perfusion, particularly in the kidneys, has been demonstrated through a substantial sample size, with a defined DO2i threshold set at 270 mL/min/m^2^ [16]. The disparity in DO2 levels observed during CPB was statistically significant between the study groups; however, it is noteworthy that both groups maintained a DO2 level exceeding 270 mL/min/m^2^. Patients within the study group exhibited a statistically significant reduction in creatinine levels immediately following surgery, 24 and 48 hours post-CPB; however, the variation in creatinine levels was not statistically significant at the 12-hour interval after surgery.

The positive results observed can be ascribed to two main elements: the increased CI and the integration of pulsatile flow into the CPB management strategy.

HCT serves as an essential factor in the evaluation of DO2, in conjunction with cardiac output or the blood flow provided by the HLM [1]. Adequate DO2 during CPB is essential for maintaining normal aerobic metabolic processes and for mitigating the risk of lactate accumulation, which can occur as a result of diminished cellular perfusion or oxygen deprivation. In this study, we ensured that the HCT levels were kept between 25% and 27% to effectively assess the effects of different CI.

The findings of this research demonstrate that the group subjected to a higher CI experienced a significant decrease in lactate levels during and after the surgical procedure, in contrast to the control group (*P* < 0.05). Our experience with CPB management indicates that a CI of 2.4 L/min/m^2^ is inadequate for ensuring effective organ perfusion. Research has shown that a DO2 value exceeding 300 mL/min/m^2^ is essential to prevent adequate organ perfusion, but this is not attainable with a CI of 2.4 L/min/m^2^ [17, 18]. Furthermore, the regulation of flow can be conducted autonomously or tailored to align with the metabolic requirements of the patient, which may be influenced by factors such as haemodilution, temperature fluctuations during cooling and warming, systemic inflammatory response syndrome, and cellular acidosis, among others, and can persist throughout CPB [19]. Consequently, it has been assessed that a CI of 2.4 L/min/m^2^ is inadequate for optimal physiological function.

The results obtained by Condello and Clingan align with the outcomes of the current study, suggesting that maintaining a cardiac index of 3 L/min/m^2^ is vital for mitigating hyperlactatemia, as it allows the DO2 to exceed 300 mL/min/m^2^ [1, 10].

In the framework of the study group, we also utilized PF to improve organ perfusion subsequent to the elevation of CI. The beneficial effects of combining elevated CI and PF may stem from multiple physiological mechanisms. Pulsatile flow is believed to enhance endothelial function through increased shear stress, promoting nitric oxide release, which facilitates vasodilation and improves microcirculation [20, 21]. Additionally, the enhanced CI contributes to a sustained oxygen delivery that exceeds metabolic demand, reducing the likelihood of anaerobic metabolism and subsequent lactate accumulation [13, 22]. These mechanisms collectively support more stable perfusion pressure and reduced ischemic risk across vital organs.

Studies demonstrate that the physiological features of arterial pulsatile flow can be successfully mimicked by utilizing the heart-lung machine (HLM) pulsatile flow configurations [8]. Quality of the pulsatile flow was assessed using EEP. Modifications to the width and frequency settings were implemented to ensure that the EEP exceeded the MAP by 15% [21]. Furthermore, it is important to note that in the recent quantification of pulsatile flow, the EEP is utilized to evaluate the quality of pulsatile flow by calculating the area beneath the arterial pressure curve and the area beneath the pump flow curve during the pulsatile cycle. In this context, it is essential to maintain the EEP value at 10–15 mmHg above the MAP [4]. Adjustments to pulsatile parameters – specifically width and frequency – were made manually approximately every 15–20 min, or earlier if the EEP-to-MAP ratio dropped below the 15% target threshold. These adjustments were individualized, depending on patient-specific hemodynamic parameters such as MAP fluctuations, systemic vascular resistance, and arterial line pressure readings. Typically, an increase in vascular resistance or a MAP drop necessitated a change in pulse width or frequency. EEP was calculated by the external mobile app using the area under the curve of the pulsatile waveform.

We propose that an increased cardiac index in conjunction with the application of pulsatile flow could facilitate a progressive improvement in organ perfusion throughout cardiopulmonary bypass for CABG patients.

This research provides a novel perspective on evaluating organ perfusion through the modification of cardiac index, while concurrently utilizing pulsatile flow techniques.

Furthermore, we assessed a range of additional complications linked to CPB. The participants in the study group exhibited a reduced requirement for inotropic support, attributed to the elevated CPB flow observed when MAP decreased, particularly in conjunction with the application of pulsatile flow (*P* < 0.05).

## Study limitations

As previously noted, this research represents the inaugural investigation into the integration of pulsatile flow with enhanced CI within the domain of perfusion science. Consequently, we opted to carry out a pilot study involving a minimal number of participants in order to evaluate potential complications. Furthermore, it was crucial to investigate supplementary factors, including plasma-free haemoglobin, to determine the likelihood of red blood cell lysis, alongside particular biomarkers such as interleukin-18 to assess sufficient kidney perfusion. We expect to undertake a more targeted study in the future.

We chose to study the effect of enhanced CI in combination with pulsatile flow (PF) based on prior literature indicating that CI levels above 2.4 L/min/m^2^ are associated with improved DO2i and reduced hyperlactatemia. A third group with only high CI and no pulsatile flow was considered; however, due to institutional limitations and the pilot nature of the study, we focused on the combined impact to assess feasibility. Future studies should evaluate CI and PF independently and in combination to isolate their individual effects.

## Conclusions

We conclude that a CI exceeding 2.4 L/min/m^2^, when paired with pulsatile flow, has the potential to enhance both intraoperative and postoperative parameters. This integrative strategy has the capacity to mimic the physiological features of pulsatile circulation, even in the absence of physiologic pulsatility within the arterial line. Additional research is required to evaluate this combination in order to either validate or refute the proposed approach to vital organ perfusion during CPB.

## Data Availability

Access to the data can be obtained from the corresponding author upon a reasonable request, contingent upon the approval of the institutional review board at the University of Baqyiatallah.
